# Application of a novel multi-segment foot model to assess foot function in children and adolescents with juvenile idiopathic arthritis

**DOI:** 10.1186/1757-1146-4-S1-O20

**Published:** 2011-05-20

**Authors:** Gordon J Hendry, Janet Gardner-Medwin, Debbie E Turner, Jim Woodburn

**Affiliations:** 1 School of Biomedical and Health Sciences, University of Western Sydney, Sydney, Locked Bag 1797, Australia; 2 Glasgow Caledonian University, Glasgow, G4 0BA, UK; 3 University of Glasgow, Glasgow, G12 8QQ, UK

## Background

Foot and ankle disease has been associated with impairments and disability in JIA, and altered gait patterns have been described. However, the extent to which disease-related foot impairments impact on localised biomechanical foot function remains unknown. The aim of this study was to compare biomechanical foot function between patients with JIA and healthy participants.

## Methods

14 JIA patients with a history of foot disease and 10 healthy controls were recruited. Participants underwent 3D gait analysis, plantar foot pressure and spatio-temporal gait evaluation. Each participant completed the Juvenile Arthritis Foot Disability Index (JAFI) and CHAQ. US scores for effusions and synovial hypertrophy, tender/swollen foot joint counts, and deformity scores were recorded to assess localised disease activity and impairments. Mean differences and 95% confidence intervals (CI) were calculated using the *t* distribution.

## Results

In the JIA group there were low but variable levels of localised disease activity indicated by low median (range) scores for tender [0 (0-4); 0 (0-5)], and swollen foot joints [0 (0-3); 0 (0-3)], US effusions [2 (0-6); 3 (0-7)] and synovitis [0 (0-3); 0 (0-2)]. Mild to moderate foot related impairments [JAFI; 1 (0-3)] and foot deformity scores [forefoot; 2 (0-7), 0.5 (0-5); rearfoot; 2.5 (1-5); 3.5 (2-5)] were observed. No significant differences were observed between group means for all core variables except right peak mid-foot dorsi-flexion [mean (95% CI) difference -3.04° (-5.79, -0.30)]. There were trends towards reduced walking velocity [mean difference 7.81cm/s (-8.26, 23.88)], greater variability in mid-foot contact area (cm^2^) [mean (SD) 19.77 (11.63); 19.04 (12.27) JIA group versus 19.31 (5.29), 19.01 (4.46) controls], and lower peak forefoot pressures (kPa) in the JIA group [mean difference 59.33 (-58.38, 177.03); 13.36 (-121.02, 147.73)].

## Conclusions

At the group level, foot function appeared normal despite moderate levels of self reported impairment and disability in participants with JIA. At the individual level detailed changes in foot function relevant to flat-footedness or highly arched foot types were detected. Preliminary findings suggest that such foot types may be associated with abnormal foot segment rotations. Further study is required to evaluate foot biomechanics in clinically meaningful homogeneous subgroups of JIA patients.

